# Risk of latent tuberculosis in at-risk children with rheumatic diseases

**DOI:** 10.1186/1546-0096-9-S1-P218

**Published:** 2011-09-14

**Authors:** SC Lim, JE Munro, RC Allen, N Curtis, JD Akikusa

**Affiliations:** 1 Rheumatology Unit, Royal Children’s Hospital, Melbourne, Australia; 2 Infectious Diseases Unit, Department of General Medicine, Royal Children’s Hospital, Melbourne, Australia; 3 Department of Paediatrics, University of Melbourne, Australia

## Background

The risk of developing latent TB infection (LTBI) following exposure to active tuberculosis (TB) in children with rheumatic diseases on immunosuppressive therapy is not well established.

## Aim

To determine the risk of developing LTBI in children with rheumatic diseases following inadvertent exposure to active TB in the course of their clinical care.

## Methods

Data from TB screening by tuberculin skin test (TST) and interferon gamma release assay (Quantiferon TB Gold In tube, (IGRA)) involving at-risk children with rheumatic disease was reviewed. Exposed patients were considered to be at moderate risk of LTBI if they were under 5 years of age, or receiving conventional DMARDs or corticosteroids > 0.5 mg/kg/day, and at high risk if they were receiving biologic DMARDS. High risk patients also had chest X-rays.

## Results

Results from 55 children were analyzed; 13 (23.6%) in the high risk category and 42 (76.4%) in the moderate risk category. The duration of exposure to the index case ranged from 30 to 120 minutes. Two children in the high risk group on Etanercept tested positive for TST (negative IGRA). All patients tested negative for IGRA. A positive test result did not correlate with the degree of immunosuppression or cumulative exposure. No patients had symptoms or signs suggestive of active TB and all chest radiographs were normal.

## Conclusion

The risk of testing positive for LTBI was low in this cohort of children, even in those on potent immunosuppressants. The sensitivity of TST and IGRA in this situation remains unknown.

